# Deep sequencing of BCR heavy chain repertoires in myalgic encephalomyelitis/chronic fatigue syndrome

**DOI:** 10.3389/fimmu.2025.1489312

**Published:** 2025-02-17

**Authors:** Audrey A. Ryback, Graeme J. M. Cowan

**Affiliations:** Institute of Immunology and Infection Research, School of Biological Sciences, University of Edinburgh, Edinburgh, United Kingdom

**Keywords:** ME/CFS, BCR repertoire sequencing, AIRRseq, MS, myalgic encephalomyelitis

## Abstract

Myalgic encephalomyelitis/chronic fatigue syndrome (ME/CFS) is a common and debilitating chronic illness of unknown aetiology. Chronic infection and autoimmune responses have been proposed as two mechanisms that potentially underlie the pathogenesis of ME/CFS. To explore these disease hypotheses, we characterised the antigen-specific receptors of B cells using adaptive immune receptor repertoire sequencing. We compared the B-cell receptor (BCR) repertoires of 25 patients with mild/moderate ME/CFS, 36 patients with severe ME/CFS, 21 healthy controls, and 28 patients with multiple sclerosis (MS) to identify signatures of infection or autoimmune responses. ME/CFS patients did not display increased clonality or differential somatic hypermutation compared to healthy controls and patients with MS. One of two immunoglobulin heavy variable (IGHV) genes, IGHV3-30, reported to be increased in ME/CFS patients in a previous study, was replicated in patients with mild/moderate disease in our cohort. However, there was no evidence of ongoing adaptive responses in IGHV3-30 repertoires from mild/moderate ME/CFS patients with increased IGHV3-30 usage. There were no detectable repertoire signatures associated with infection or autoimmunity in repertoires from ME/CFS patients, but we observed skewing of the ratio of IgM to IgG BCRs in patients with mild/moderate ME/CFS, a preliminary finding that presents an opportunity for follow-up work.

## 1 Introduction

Myalgic encephalomyelitis/chronic fatigue syndrome (ME/CFS) is a disease of unknown aetiology and pathophysiology that affects an estimated 250,000–390,000 people in the UK (1, 2). Individuals with ME/CFS experience, on average, greater disability than patients with type 2 diabetes, congestive heart failure, multiple sclerosis (MS), and most cancers (3). The onset of ME/CFS symptoms is preceded by an infectious episode in more than 60% of patients (4–6), and infectious mononucleosis is the most commonly reported infection experienced prior to onset of ME/CFS (4). Some research findings suggest an autoimmune or immune-mediated component to the disease (7, 8). Elevated levels of autoantibodies against beta-adrenergic receptors, which are also found in healthy individuals and form part of the “natural” autoantibody repertoire (9), have been reported in several studies (10, 11). Furthermore, HLA associations (HLA-C*07:04 and HLA-DQB1*03:03) have been found in one study of >4,000 ME/CFS patients and matched controls (12).

B-cell receptors (BCRs) determine the antigen specificity of each B cell. BCR-antigen binding and the affinity of the binding play a critical role in the activation, maturation, selection, and differentiation of B cells from naive to memory or plasma cells (13, 14). Furthermore, evidence of ongoing or recent adaptive responses and previous germinal centre reactions can be observed in the BCR repertoire, as these processes skew the abundance of different BCRs in the repertoire and introduce mutations into BCRs during affinity maturation. BCR repertoire sequencing involves sequencing BCRs from a large number of cells from an individual and has demonstrated altered features of BCR repertoires in infection (15), vaccination (16), ageing (17), and immune-mediated and autoimmune diseases (18). BCR repertoire sequencing in ME/CFS and healthy controls (HCs) previously identified increased use of several Immunoglobulin Heavy Chain V (IGHV) gene segments in ME/CFS patients: IGHV-3-30 and IGHV3-30-3 gene usage was elevated in ME/CFS, particularly in patients who reported an infectious onset to their illness (19). The authors suggest this IGHV gene signature may represent a shared infection-elicited response among those ME/CFS patients. This result putatively fits a signature of dysregulated IGHV3-30/IGHV3-23 antibody detected by plasma proteomic profiling (20).

V gene usage in the naive BCR repertoire is primarily genetically determined by individuals’ diverse germline repertoires—the naive BCR repertoire is highly similar in identical twins (21)—but trends in V gene or allele usage are observed across populations (22, 23). In acute infection or in response to vaccination, activated B cells undergo clonal expansion, which biases the composition of the V gene repertoire towards the V genes present in the expanded clonotypes. Biased V gene usage has been particularly well characterised in influenza infection, where IGHV1-69 is frequently a feature of broadly neutralising influenza-specific antibodies to the haemagglutinin (HA) stem (24) mediated by hydrophobic residues encoded by the IGHV gene (25). Responses to infection are usually also accompanied by increased somatic hypermutation, as activated B cells undergo affinity maturation and clonal selection, and class switch recombination from IgM to IgG, IgA, or IgE (26–28).

In autoimmune and immune-mediated diseases, V gene biases due to antigen-driven clonal expansion are sometimes observed, but V gene skews may also occur due to defects in tolerance (29). IGHV4-34 is a V gene known to give rise to self-reactive BCRs that can bind red blood cell antigen and other self-antigens and commensal bacteria (30, 31). Increased use of IGHV4-34, particularly un-mutated IGHV4-34, has been observed in repertoires from patients with systemic lupus erythematosus (SLE), Crohn’s disease, and eosinophilic granulomatosis with polyangiitis (18, 32, 33) and is thought to reflect aberrations in B-cell tolerance checkpoints (33). *N*-glycosylation sites are rare in the germline IG repertoire (found only in IGHV4-34, IGHV5-10-1, IGHV1-8, IGLV3-12, and IGLV5-37), but are more frequent in the BCR repertoires of patients with immune-mediated diseases (34–36). *N*-glycosylation in the variable regions of BCRs have been shown to enhance B-cell activation upon antigen binding (34). In rheumatoid arthritis (RA) and SLE, BCR repertoires are additionally skewed towards IgG BCRs with fewer mutations and are hypothesised to be the result of defects in affinity maturation or extra-follicular B-cell activation (32, 37). Thus, defects in B-cell tolerance and selection can skew the developing BCR repertoire in immune-mediated disease.

## 2 Materials and methods

### 2.1 Ethics statement

Samples were obtained from the CureME Biobank, and ethical approval was granted by the University College London Biobank Ethical Review Committee (RFL B-ERC) (ref. EC.2018.006) to use the samples for both a TCR study (38) and this study.

### 2.2 Samples

Samples were obtained from the CureME Biobank as frozen PBMC stocks isolated from blood and pre-processed as described in Dibble et al. (38). ME/CFS patients were included if they had a previous diagnosis of ME and met either the Canadian Consensus Criteria or the Fukuda Criteria. Severe patients were sampled in their homes. Samples were processed by Systems Biology Laboratory (SBL) in Oxfordshire. CD8, CD4, and γ*δ* T cells were obtained by MACS sorting, and the remaining cells were stained for CD19+ B cells and snap frozen as cell pellets.

### 2.3 BCR library prep

Blinded T cell-depleted samples were obtained from SBL. The library preparation strategy was adapted from Turchaninova et al. (39). RNA extraction was performed using the Zymo Quick-RNA Miniprep Plus Kit (Zymo Research#R1058) as per the manufacturer’s instructions, and the DNA digestion step was performed. cDNA synthesis was performed using the Takara SMARTScribe cDNA synthesis kit (#639538) using constant region-specific primers (“R1 primers”, see **Supplementary Table S1**). R1 primers were mixed 1:1, and 2 µL of 10 µM working stock was added to 8 µL of RNA to 0.2-mL reaction tubes (Corning #CLS3745) and incubated for 2 min at 70°C followed by 42°C for 1–3 min to allow the R1 primers to anneal. cDNA Synthesis Mix (12 µL; see **Supplementary Table S2**) was added directly to each sample tube still in the thermocycler and mixed by pipetting. Reactions were incubated for 60 min at 42°C, followed by 70°C for 10 min. One microliter of Uracil DNA Glycosylase (5 U/µL) (NEB #M0280L) was added directly to each reaction and incubated for 15 min at 37°C. For PCR1, 2 µL of freshly synthesised cDNA was added to PCR1 mastermix (see **Supplementary Table S3**) and PCR cycling was performed as follows: Initial denaturation at 98°C for 2 min, followed by 18 cycles of 98°C for 10 s, 72°C for 15 s, and 72°C for 25 s, and a final extension at 72°C for 4 min. PCR 2 was prepared as described in **Supplementary Table S4**, and PCR cycling conditions were as follows: Initial denaturation at 98°C for 2 min, followed by 18 cycles of 98°C for 10 s, 72°C for 15 s, and 72°C for 25 s, and a final extension of 72°C for 4 min.

### 2.4 Sequencing

BCR libraries were run on a 2% agarose gel, with 0.5× SYBR-Safe and a band between 400 and 800 bp was extracted using the NEB Monarch DNA Gel Extraction kit (catalogue #T1020S). Libraries were submitted to Genewiz (Azenta) for two runs of sequencing on the Illumina Miseq v3, with paired-end asymmetric sequencing (400 cycles in read 1, 200 cycles in read 2) with custom read 1, read 2, and index primers (**Supplementary Table S1**).

### 2.5 Sample exclusions

Samples were blinded when performing library preparations and data pre-processing, and decisions regarding sample exclusions were made prior to unblinding. Samples with less than 1,500 UMI-labelled productive BCR sequences were excluded. One sample was additionally excluded because the individual (an ME/CFSmm patient) had a highly aberrant repertoire: >60 million B cells, 96% of their BCRs used IGHV4-34, and 90% of the BCRs had an identical complementarity-determining region 3 (“CARVGAHYYYYYMDVW”).

### 2.6 Data analysis

Fastq files were pre-processed using tools in the Immcantation suite (Version 0.4.5) in pRESTO (40) to remove low-quality reads and pair reads, extract UMIs, and build consensus sequences from reads with the same UMIs. Consensus sequences were then aligned to IMGT reference databases of IGHV, IGHD, and IGHJ genes using IgBlast (version 1.17.0) via the Change-O wrapper (41). Non-productive BCRs were excluded from analysis. All analyses were performed in Python (version 2.7.5) using custom scripts in base Python and the pandas package (version 1.3.5) (42). Seaborn (version 0.12.0) (43) and matplotlib (version 3.5.3) (44) packages were used for plotting unless specified. Analyses relating to V3-30 high ME/CFSmm repertoires and analyses examining IgM to IgG ratios were performed *post-hoc* and were not included in the original analysis plan.

For diversity/clonality analysis, CDR3 amino acid sequences were subsampled to 1,000 UMIs, and the Gini Index of Inequality and Shannon entropy were calculated. This process was repeated over 1,000 iterations and diversity/clonality was averaged across all iterations for each repertoire. Diversity/clonality was calculated either on unique complementarity-determining region 3 (CDR3s) or, for diversity analyses of clonotypes shown in the network diagrams, on pre-clustered clonotypes. Diversity/clonality indices were calculated as follows:

#### 2.6.1 Gini inequality


$$G=\frac{\sum_{i=1}^{S}(2i-S-1)\cdot P_{i}}{n\cdot \sum_{i=1}^{S}P_{i}}$$

where *S* is the number of species and $P_{i}$ is the proportion each species makes up of the population.

#### 2.6.2 Shannon entropy


$$H=\sum_{i=1}^{S}-\left(P_{i}\;\times \;lnP_{i}\right)$$

where *S* is the number of species and $P_{i}$ is the proportion each species makes up of the population.

*N-*glycosylation sites were identified by first translating the BCR nucleotide sequences and then identifying *N*-glycosylation motifs based on amino acid sequence (motifs N-X-S/T where X can be any amino acid except for proline).

Somatic hypermutation was quantified by counting mismatches between BCR nucleotide sequences and the germline sequences they were aligned to. Mutation frequency was calculated by dividing the number of mutations in a sequence by the nucleotide length of that sequence.

### 2.7 Statistical testing

Linear regression, Kruskal–Wallis and Mann–Whitney *U*-tests were performed in Python using the scipy.stats package. Mann–Whitney *U*-tests were performed for V gene usage testing as in Sato et al. (19) and Bonferroni correction was applied. Kruskal–Wallis tests were used to compare other parameters, unless specified. *Post-hoc* tests were performed using Dunn’s test in the scikit_posthocs package (45). *p*-values with and without multiple testing correction (Holm–Sidak correction) were reported. Linear models and generalised linear models were run using the lm and glm packages in base R [R version 4.2.0 (46)]. For receiver operating characteristic (ROC) analysis, data were split into a training (60%) and test set (40%), with each group being represented in the same proportions as in the original data set. ROC analysis was conducted in R (47), using a generalised linear model to perform multivariate logistic regression and results were plotted using ggplot2 (48).

### 2.8 Network diagrams

BCRs were clustered into “clonotypes” using all BCR sequences from all individuals to allow for the identification of shared clonotypes between different individuals. Sequences sharing the same V and J gene call, CDR3 length, and CDR3 amino acid sequences within an edit distance of 0.15 substitutions per aa across the whole CDR3 region were clustered using hierarchical clustering in scikit.learn (45). IGHV3-30 repertoires were subset to 10 individuals for each group to allow for visual comparison and randomly subsampled to 5,000 UMIs per group. A similarity matrix was constructed for IGHV3-30 clonotypes from ME/CFSmm individuals with high IGHV3-30 usage, and for IGHV3-30 clonotypes from HCs. UMI-identified BCRs assigned to the same clonotype were connected by an edge and were plotted in Gephi (49).

## 3 Results

### 3.1 Study design

To investigate whether features of the BCR repertoire from mild/moderate and from severe ME/CFS patients differed from HCs and patients with MS, BCR repertoire sequencing was performed on a total of 124 T cell-depleted PBMC samples from the CureME Biobank. A total of 110 samples had sufficient depth for analysis (>1,500 UMI-labelled cDNA transcripts) and passed quality control checks, comprising 25 patients with mild/moderate ME/CFS (ME/CFSmm), 36 patients with severe ME/CFS (ME/CFSsa), 21 healthy controls (HCs), and 28 MS controls (MS). The CureME Biobank defines “mild” disease as approximately a 50% reduction in pre-illness activity levels, “moderate” as mostly housebound, and “severe” as mostly bed bound. Patients with severe ME/CFS were sampled at home. Starting B-cell numbers varied widely across samples (2,840–1,590,300, median = 56,790 CD19+ cells) but did not vary significantly between groups (**Supplementary Figure S1B**). Repertoire sequencing of IgM and IgG BCRs was performed, adapted from Turchaninova et al. (2016) (primer and sequencing strategy shown in **Supplementary Figure S1A**).

The primary analysis plan set out to:

1. Test differences in repertoire diversity and clonal expansion between the two ME/CFS groups and the control groups.
2. Examine differences in V gene usage patterns between the two ME/CFS groups and the control groups.
3. Replicate the analysis reported by Sato et al. (19) to test whether ME/CFS patients had increased IGHV3-30 and IGHV3-30-3 gene usage.
4. Use the six features of the repertoire (IGHV3-30, IGHV3-30-3, IGHV3-49, IGHV1-3, IGHD1-26, and IGHJ6 usage) used to predict ME/CFS status in Sato et al. (19), to train a classifier that differentiates between ME/CFS and HCs in our cohort.
5. Quantify somatic hypermutation in both IgM and IgG isotypes, and quantify the frequency of *N*-glycosylation sites in BCRs.

### 3.2 There are no differences in repertoire diversity between ME and control groups

**Figure 1:**
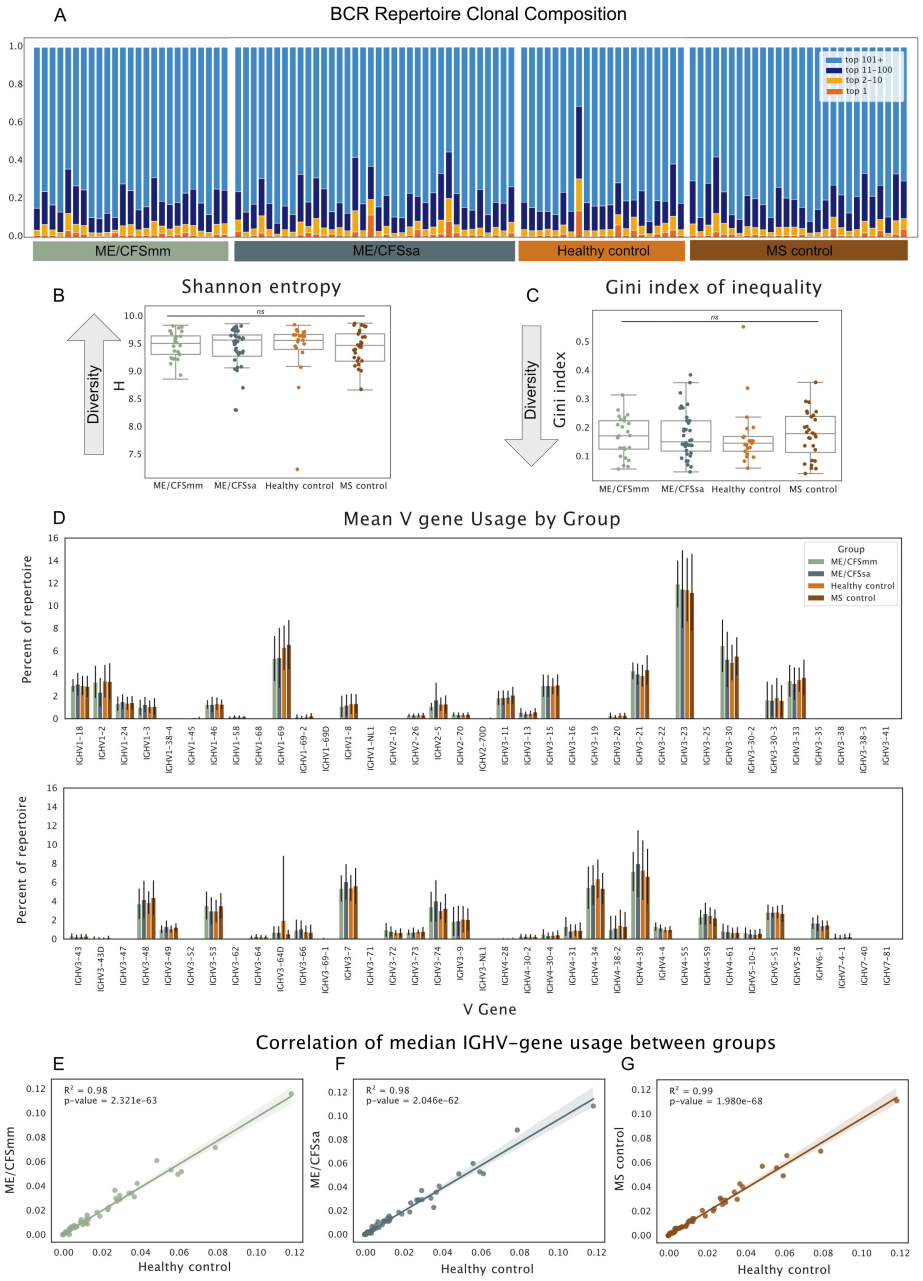
**(A)** Proportion of the repertoire occupied by the top 1, 2–10, 11–100, and 101+ most abundant BCRs. Each bar represents one individual’s repertoire. **(B)** Shannon entropy *H*. for each repertoire. **(C)** Gini Index of Inequality for each repertoire. For diversity metrics, repertoires were downsampled to 1,000 UMIs prior to calculation of diversity metrics. Repertoires were re-sampled over 1,000 iterations and diversity metrics were averaged across the iterations to generate the estimates. Significance was tested in B and C with Kruskal–Wallis test (alpha < 0.05; ns, not significant). **(D)** Mean V gene usage by disease group shown for all V genes. Error bars represent standard deviation around mean. Median V gene usage was calculated for each V gene in all four groups. Each dot represents the median proportion of a given V gene, and their correlations are shown for **(E)** ME/CFSmm and healthy controls, **(F)** ME/CFSsa and healthy controls, and **(G)** MS controls and healthy controls.

An ongoing adaptive B-cell response to self- or foreign antigen will usually be detected as clonal expansion and skewing of the BCR repertoire towards B cells with a particular CDR3, or similar CDR3 amino acid motifs, during the acute phase of the adaptive response (13). We characterised the composition of each individual repertoire by binning BCRs according to whether their CDR3s were the top 1, top 10, top 100 or top 100+ most abundant in the repertoire. Repertoire composition varied substantially between the individuals, but no consistent differences were observed between groups (**Figure 1A**). To quantify differences in repertoire composition relating to clonality (dominance of a particular clone) and diversity (“evenness” of the repertoire), we calculated two commonly used clonality and diversity metrics: Shannon entropy and the Gini Index of Inequality. While the Gini Index is sensitive to even small clonal expansions, Shannon entropy is influenced more by larger expansions as well as the total number of unique BCRs in the repertoire. No significant difference in diversity or clonality was found between the groups using either of these diversity metrics (**Figures 1B, C**). This suggests that there were no marked differences in clonal expansion in any of the groups.

### 3.3 Global V gene usage is comparable among groups

To examine patterns of V gene usage across the repertoires, we next quantified the proportion that each V gene made up of a given repertoire and compared the average V gene usage for each group. Patterns of V gene usage were broadly consistent across groups (**Figure 1D**). As is found in other studies, V genes of the IGHV3 family, in particular IGHV3-23, were the most commonly used V genes in repertoires, followed by the IGHV4 gene family. Median V gene usage was highly correlated between groups (*R* ^2^ = 0.98–0.99), suggesting that there were no global differences in V gene usage between ME/CFS patients and HCs, and MS and HCs (**Figures 1E–G**).

### 3.4 Increased IGHV3-30 gene usage replicates in ME/CFSmm patients

**Figure 2:**
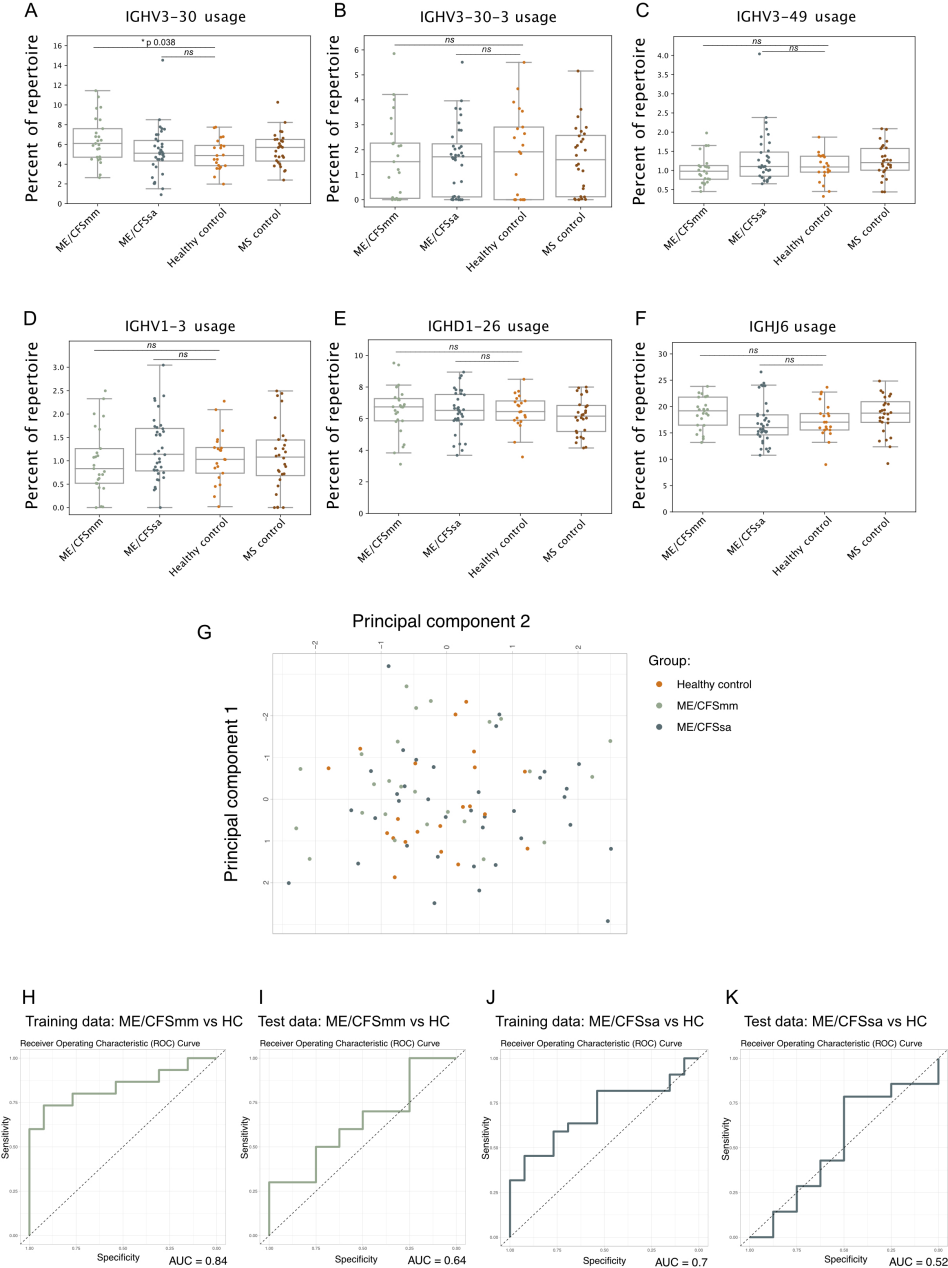
Strip plots of six distinguishing features of the ME/CFS repertoire in our cohort that were previously identified in Sato et al.: **(A)** IGHV3-30, **(B)** IGHV3-30-3, **(C)** IGHV3-49, **(D)** IGHV1-3, **(E)** IGHD1-26, and **(F)** IGHJ6 gene usage. Percentage of the repertoire made up of the V, D, or J gene segment was tested using Mann–Whitney *U*-test between ME/CFSmm and healthy controls and ME/CFSsa and healthy controls, and corrected for multiple testing using Bonferroni adjustment (alpha < 0.05; ns, not significant). **(G)** Principal component analysis of the six V, D, and J gene features for healthy controls, ME/CFSmm, and ME/CFSsa. Receiver operating characteristic curves shown for Training and Test data using logistic regression model using the six repertoire features shown in **(A–F)** to predict ME/CFSmm **(H, I)** or ME/CFSsa status **(J, K)**.

Next, we sought to replicate the specific findings of increased IGHV3-30 and IGHV3-30-3 gene usage in patients with ME/CFS compared to controls reported in Sato et al. (19). In our cohort, IGHV3-30 gene usage was increased in ME/CFSmm patients compared to HCs (*p* = 0.038, Bonferroni corrected, **Figure 2A**). The effect size of this difference in IGHV3-30 gene usage was moderate (Cohen’s *D* = 0.70) but similar between the two studies (Cohen’s *D* estimated from data shown in Sato et al., 19: 0.69). IGHV3-30-3 gene usage did not differ among groups (**Figure 2B**). However, IGHV3-30 and IGHV3-30-3 are very closely related V gene families: IGHV3-30*4 and IGHV3-30-3*3 share identical nucleotide sequences in the international ImMunoGeneTics information system (IMGT) reference database. On a neighbour-joining phylogenetic tree, alleles of IGHV3-30 and IGHV3-30-3 do not belong to distinct monophyletic groups (**Supplementary Figure S2A**). It is therefore likely that IGHV3-30 and IGHV3-30-3 BCRs are highly similar and may share similar antigenic targets. Differential allele usage or differences in how alleles were called in our cohort and the repertoire from Sato et al. (19) may have resulted in only one of the IGHV gene signatures replicating. Comparing combined IGHV3-30 and IGHV3-30-3 gene usage between groups showed a similar pattern to IGHV3-30 usage only, but IGHV3-30/IGHV3-30-3 usage in mild/moderate ME patients was no longer statistically significant (**Supplementary Figure S2B**).

### 3.5 Previously reported BCR repertoire features do not predict ME/CFS cases in our study

Sato et al. (19) trained a logistic regression model on six features of the BCR repertoire that was able to distinguish between ME/CFS patients and HCs with high sensitivity and specificity (AUCs of 0.85–0.9). We attempted to replicate this analysis as described in the paper using the proportions of IGHV3-30, IGHV3-30-3, IGHV1-3, IGHV3-39, IGHD1-26, and IGHJ-6 (**Figures 2A–F**) in the repertoire. Notably, IGHJ-6 usage showed a trend towards being increased in ME/CFSmm patients, but was not statistically significant (*p* corrected = 0.11, **Figure 2F**). Principal component analysis did not suggest separation of ME/CFSmm, ME/CFSsa, and HCs based on the six repertoire features (**Figure 2G**). The training data had AUCs of 0.84 and 0.70 (**Figures 2H, J**), and the model performed poorly when predicting cases on the test data for mild/moderate (AUC 0.64) and severe ME/CFS (AUC 0.52) (**Figures 2I, K**). Combining both ME/CFS groups did not improve the model’s performance (data not shown). In our cohort, these repertoire features were not able to predict ME/CFS cases. This could be explained by genetic differences between the two populations that the cohorts were sampled from (Japan and the United Kingdom) as V, D, and J gene usage can differ among population groups and elicit different immune responses to the same antigen (23, 25).

### 3.6 Features of IGHV3-30 BCRs do not differ between ME/CFSmm patients and healthy controls

**Figure 3:**
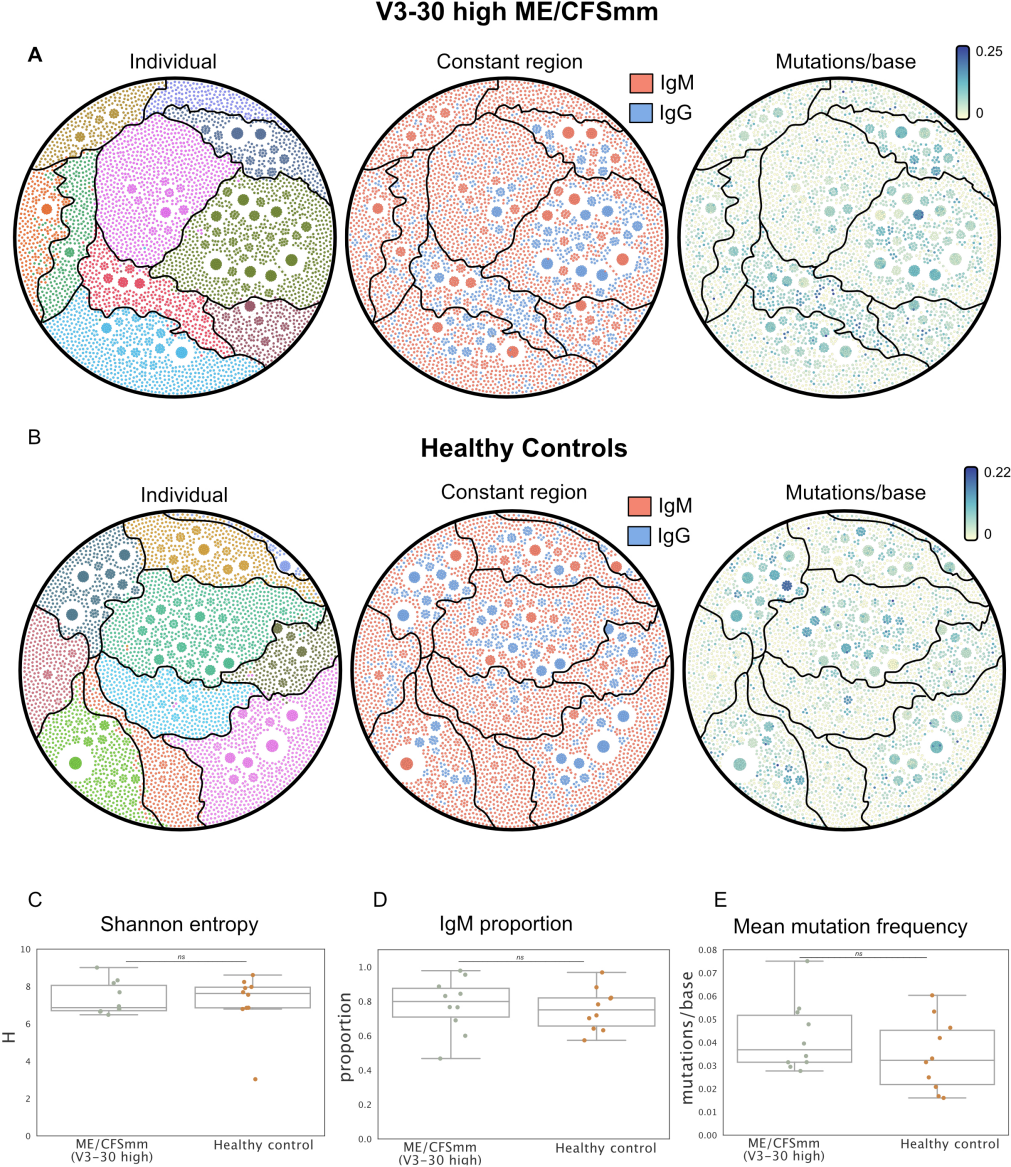
Network diagrams showing IGHV3-30 BCR repertoires from **(A)** ME/CFSmm with ≥1 standard deviation IGHV3-30 gene usage (10 individuals), and **(B)** 10 healthy controls selected at random. Each node corresponds to a unique BCR transcript and edges represent BCRs that belong to the same clonotype. Network diagrams are coloured by individual (left), constant region call (centre), and mutation frequency (right). **(C)** Shannon entropy, **(D)** IgM proportion, and **(E)** average mutation frequency were calculated for data shown in **(A, B)** (alpha < 0.05; ns, not significant).

We next looked to explore whether the increased usage of IGHV3-30 in ME/CFSmm patients might reflect B-cell responses driven by a common antigen. If this were the case, we might expect (1) increased clonality, (2) increased somatic hypermutation, and (3) increased class switching from IgM to IgG in IGHV3-30 from ME/CFSmm patients. To test these *post-hoc* hypotheses, we selected ME/CFSmm patients who had IGHV3-30 gene usage >1 standard deviation than the mean healthy control IGHV3-30 levels (10 patients), and sampled the same number of HCs at random. To allow for minor differences in CDR3 sequence due to somatic hypermutation or differential allele usage, we analysed BCR “clonotypes” rather than unique CDR3s. BCR clonotypes were assigned to BCRs with the same V and J gene call, CDR3 length, and CDR3s within an amino acid edit distance of 0.15 amino acid substitutions across the entire CDR3. We plotted the IGHV3-30 clonotype repertoires from ME/CFSmm patients (**Figure 3A**) and HCs (**Figure 3B**) as network diagrams and annotated them by sample (left), constant region (centre), and mutation burden (right). We also quantified Shannon entropy (**Figure 3C**), isotype usage (**Figure 3D**), and mutation frequency (**Figure 3E**). These subsetted repertoires did not differ between ME/CFSmm and HCs based on any of the above features. Additionally, there were no clusters of shared clonotypes between the patients. These data did not suggest more shared or convergent clonotypes between individuals with high IGHV3-30 gene usage in the ME/CFSmm group, compared to healthy individuals.

### 3.7 Somatic hypermutation profiles are similar among groups

**Figure 4:**
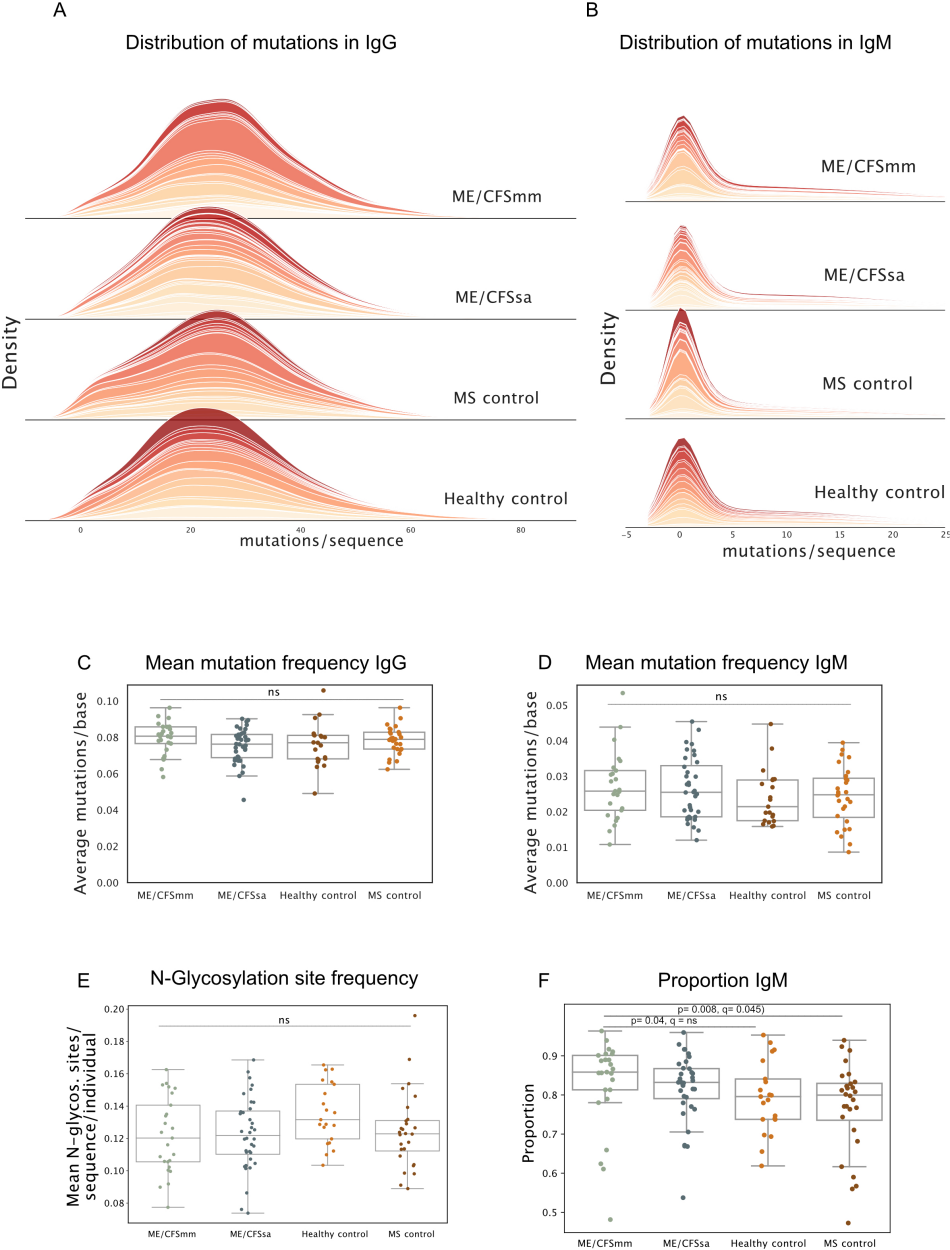
**(A)** Density plots of distribution of mutations per sequence for repertoires in each group for IgG and **(B)** IgM. Each band represents one individual. **(C)** Mean mutation frequency in IgG (mutations/base) and **(D)** IgM quantified for each repertoire. **(E)** Mean number of *N*-glycosylation sites per sequence per repertoire. **(F)** The proportion of the repertoire consisting of IgM BCRs shown for each group. Significance was tested at alpha < 0.05, using Kruskal–Wallis test, and *post-hoc* testing performed using Dunn’s test with Holm–Sidak multiple testing correction. Ns = not significant, *q*-value = *p*-value with multiple testing correction applied.

Finally, we probed additional repertoire features that are commonly skewed in chronic infection and immune-mediated disease that are affected by affinity maturation and tolerance. In chronic infection, long-term antigenic stimulation can result in an increase in mutations in BCRs, as is seen in chronic hepatitis C virus patients (50). Conversely, in some rheumatic diseases, mutations in IgG BCRs are decreased (37), and *N*-glycosylated BCRs are more common (34). We therefore quantified the number of mutations in the BCRs relative to germline and the frequency of *N*-glycosylation sites. Mutations in IgG repertoires were normally distributed, as expected (**Figure 4A**), while mutations in IgM repertoires were highly zero-inflated (**Figure 4B**) since a majority of IgM BCRs in the peripheral repertoire come from antigen-naive B cells. The average number of mismatches compared to germline per BCR sequence did not differ among the four groups for IgG (**Figure 4C**) or IgM (**Figure 4D**), although several MS patients displayed a skew towards IgG BCRs with very few mutations. Most variable regions of BCRs do not contain *N*-glycosylation sites because they are rare in germline sequences and are usually introduced by somatic hypermutation. The frequency of *N*-glycosylation motifs in BCRs did not differ among groups in our cohort, although healthy control repertoires showed a trend towards having a higher frequency of *N*-glycosylation (**Figure 4E**). Therefore, the repertoires of ME/CFS patients did not display features relating to somatic hypermutation that would suggest defects in central tolerance, clonal selection or affinity maturation, or chronic antigen stimulation.

### 3.8 IgM transcripts are over-represented in ME/CFSmm patients

Unexpectedly, the proportion of IgM BCR transcripts was significantly increased in ME/CFSmm patients compared to the other groups (**Figure 4F**) (ME/CFSmm vs. HC *p* = 0.04, ME/CFSmm vs. MS *p* = 0.008), although this analysis was not part of the original hypotheses we set out to test. Statistical significance between ME/CFSmm and healthy control groups was lost when multiple testing correction was applied, but the difference between ME/CFSmm and MS controls remained significant (*q* = 0.045). An increased ratio of IgM to IgG in ME/CFSmm patients is an exploratory result and would need to be replicated and tested by orthogonal methods (such as IgM and IgG expression on B cells by flow cytometry) to confirm its relevance. However, it could indicate increased proportions of naive B cells in the peripheral repertoire or increased IgM+ memory B cells in ME/CFSmm patients.

## 4 Discussion

In this study, we applied BCR repertoire sequencing to determine whether changes in V gene usage, diversity and clonal expansion, somatic hypermutation, and *N*-glycosylation site frequency were associated with ME/CFS. We did not observe any striking differences in these repertoire features between ME/CFS repertoires and HCs, and differences were also not observed between MS patients and HCs. We replicated a previously reported difference in IGHV3-30 gene usage in ME/CFSmm patients, but increased IGHV3-30-3 usage did not replicate in our cohort. IGHV3-30 has also previously been identified as one of two potential V genes that differed significantly in ME/CFS patients in a plasma proteomics study by Milivojevic et al. (2020). The study compared the entire plasma proteome between ME/CFS patients and HCs and described a signature of plasma IGHV3-30 or IGHV3-23 antibody (the two IGHV genes are not distinguishable by mass spectrometry), which showed a quadratic relationship: patients had either higher or lower levels of this antibody than controls. IGHV3-23 is the most abundantly used V gene, so an alternative explanation for this difference could be dysregulation of total antibody titres. Although disease severity was not explicitly reported, the cohorts included in the Sato et al. (19) paper are likely to have consisted of mostly mild/moderate ME patients since samples were collected at a hospital, which would be a significant barrier to participating in the study for severe ME/CFS patients. Severe ME/CFS patients in our cohort were house- or bed-bound and were sampled at home.

Sato et al. (19) conclude that the V gene signature they identified is consistent with a potential common antigen exposure among ME/CFS patients. Increased use of IGHV3-30 could suggest a common antigen exposure committed to B-cell memory, but we would expect this to be accompanied by either increased clonality, class switching to IgG, or mutation in IGHV3-30. We did not detect any evidence of these signatures in IGHV3-30 BCRs from ME/CFS patients with elevated IGHV3-30 usage. However, antigen specificity is determined by both heavy and light chains, and the lack of paired light chain BCR sequences is a limitation of our study. Studies in which IGHV3-30 heavy chains and their cognate light chains from ME/CFS patients are expressed as recombinant antibodies and their specificity tested against panels of target antigens (51) could reveal whether these BCRs have a common antigenic target. Examining cellular phenotypes of B cells with IGHV3-30 BCRs would also be important to determine which B-cell populations they originate from.

We probed features of the BCR repertoire commonly altered in infection and autoimmune and immune-mediated diseases. Somatic hypermutation profiles were comparable between the groups and heterogeneous among individuals, for both IgM and IgG. There was a higher proportion of IgM BCRs in the ME/CFS repertoires, particularly in ME/CFSmm patients, but this result was not included in the original hypotheses to be tested and did not survive multiple testing correction. Notably, two other studies have reported increased numbers of naive B cells in ME/CFS (52, 53), of which one also reported lower levels of isotype-switched memory B cells (53). These findings would be consistent with more IgM BCR transcripts in the peripheral repertoire. However, the ratio of IgM: IgG expression in peripheral B-cell populations has not been reported directly, and our result would benefit from being validated by other methods such as flow cytometry in future studies. Finally, the number of *N*-glycosylation sites per CDR3 did not differ between the groups. Overall, we did not observe any striking differences in the peripheral repertoire of mild/moderate ME patients or severe ME patients compared to healthy and MS controls. However, B-cell responses mostly take place in lymphoid tissues and sampling peripheral blood provides a very limited picture of adaptive immune responses. Peripheral repertoires in MS look similar to HCs, while repertoires from B cells in the cerebral spinal fluid show signs of active B-cell proliferation and selection (54, 55).

Our study is limited by the small sample size and the potentially heterogeneous populations of B cells sampled. Sorting B cells into naive, memory, and atypical B cells prior to performing repertoire sequencing would substantially improve the inferences drawn from future repertoire sequencing studies. Given the emerging evidence of altered serum antibody repertoires targeting microbiome antigens (56) in ME/CFS patients, future studies should also consider sequencing IgA, as well as IgM and IgG isotypes. Future repertoire sequencing studies in ME/CFS where repertoires from patients and controls are sampled prior to and following vaccination could shed light on whether B-cell responses to antigenic challenge are impaired in ME/CFS. Nonetheless, the use of best practices for BCR repertoire sequencing in an independent cohort make this study an important contribution to the evidence base for investigating immunological phenotypes in ME/CFS.

## Data Availability

The datasets presented in this study can be found in online repositories. The names of the repository/repositories and accession number(s) can be found below: https://www.ebi.ac.uk/ena, PRJEB83659.
