# Supplementary material for: Deep sequencing of BCR heavy chain repertoires in myalgic encephalomyelitis/chronic fatigue syndrome
Source: Front Immunol. 2025 Feb 17;16:1489312. doi: 10.3389/fimmu.2025.1489312 (PMC11872726; doi:10.3389/fimmu.2025.1489312)
Supplement: Supplementary file 1 [file DataSheet1.pdf]

Supplementary Material

1 SUPPLEMENTARY TABLES

- Table S1. PCR and Sequencing Primers (5'-3')
- Table S2. cDNA Synthesis Mix
- Table S3. PCR1 Mix
- Table S4. PCR2 Mix

2 SUPPLEMENTARY FIGURES

- Supplementary Figure S1: Library prep and sequencing strategy
- Supplementary Figure S2: IGHV3-30 and IGHV3-30-3 alleles

Table S1. PCR and Sequencing Primers (5'-3')

cDNA synthesis	
hIGG_r1	GAAGTAGTCCTTGACCAGGCA
hIGM_r1	GTGATGGAGTCGGGAAGGAAG
SMARTNNNext_12ntUMI	AAGCAGUGGTAUCAACGCAGAGTGCUNNNNUNNNNUNNNNUCTTrGrGrG
SMARTNNNext_11ntUMI	AAGCAGUGGTAUCAACGCAGAGTGCNUNNNNUNNNNUNNUCTTrGrGrG
SMARTNNNext_10ntUMI	AAGCAGUGGTAUCAACGCAGAGUGCNNUNNNNUNNNNUCTTrGrGrG
PCR 1	
M1SS_ext	GGCGAAGCAGTGGTATCAACGCAGAGTGC
hIGGE_r2	ATTGGGCAGCCCTGATTARGGGGAAGACSGATG
hIGM_r2	ATTGGGCAGCCCTGATTAGGGGGAAAAGGGTTG
PCR2	
P7+M1SS	CAAGCAGAAGACGGCATACGAGATNNNNNNGGCGAAGCAGTGGTATCAACGCAGAGT
P5+Z	AATGATACGGCGACCACCGAGATCTACACNNNNNNATTGGGCAGCCCTGATT
Sequencing primers	
hIGG_read1ext	ATTGGGCAGCCCTGATTARGGGGAAGACSGATG
hIGM_read1ext	ATTGGGCAGCCCTGATTAGGGGGAAAAGGGTTG
hBCR_read_2ext	GGCGAAGCAGTGGTATCAACGCAGAGTGC
hBCR_Index_1ext	GCACTCTGCGTTGATACCACTGCTTCGCC

Table S2. cDNA Synthesis Mix

Component	Supplier / catalogue nr	Volume per reaction
First strand buffer	Takara (#639538)	4μl
DTT	Takara (#639538)	1μl
SMARTNNNext (12uM)	IDT	2μl
dNTP (10mM)	ThermoFisher Scientific (#R0192)	2μl
SMARTscribe RTase	Takara (#639538)	2μl
RNAse inhibitor	Takara (#2313A)	1μl
RNA		8μl

Table S3. PCR1 Mix

Component	Supplier / catalogue nr	Volume per reaction
Nuclease-free water	IDT	4μl
Primer MISS_ext (10μM)	IDT	2μl
R2 primer mix (IgM+IgG combined) (10μM)	IDT	2μl
Phusion flash High Fidelity PCR Mastermix	Thermo Fisher #F548S	10μl
cDNA		2μL

Table S4. PCR2 Mix

Component	Supplier and catalogue nr	Volume per reaction
Nuclease-free water	IDT	4μl
Primer P7-SMARTamp w. sample index(10μM)	IDT	2μl
Primer BCR_P5-Rev w. sample index (10μM)	IDT	2μl
Phusion flash High Fidelity PCR Mastermix	Thermo Fisher #F548S	10μl
PCR1 product		2μl

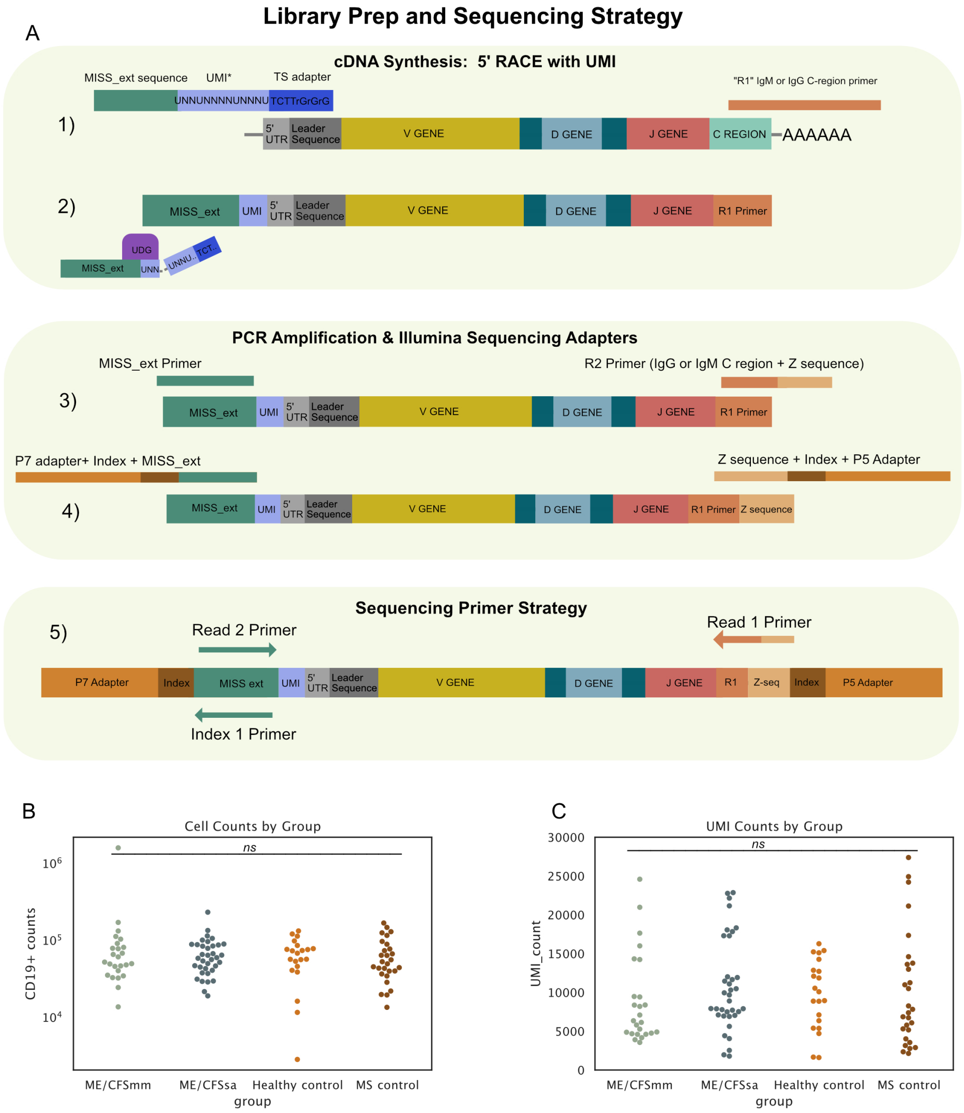

Supplementary Figure 1:
Library prep and sequencing strategy. A) cDNA synthesis was performed using **1)** IgM and IgG 3' constant region specific primers and 5' Rapid Amplification of cDNA Ends (RACE) with a "SMART" primer including a template switch motif with riboguanosine bases and a 12, 14 or 15bp UMI and a universal adapter sequence (MISS_ext) interspersed with Uracil bases so that primers **2)** can be cleaved by Uracil DNA Glycosylase after cDNA synthesis. This was followed by two round of PCR amplification: **3)** In the first round a primer for the universal MISS_ext sequence and "R2" primers for IgG and IgM were used to step further in to the constant region and introduce a universal adapter (Z sequence). **4)** In the second round of PCR two sample-specific barcoding primers were used to introduce Illumina sequencing adapters and sample indices. **5)** Custom Read 1, Read 2 and Index 1 primers were used with 400 cycles of sequencing in Read 1 and 200 cycles in Read 2. **B)** Cell counts and **C)** UMI counts for samples included in the analyses. Differences between group means were tested using a Kruskal-Wallis Test due to unequal variances between groups.

Figure S1.

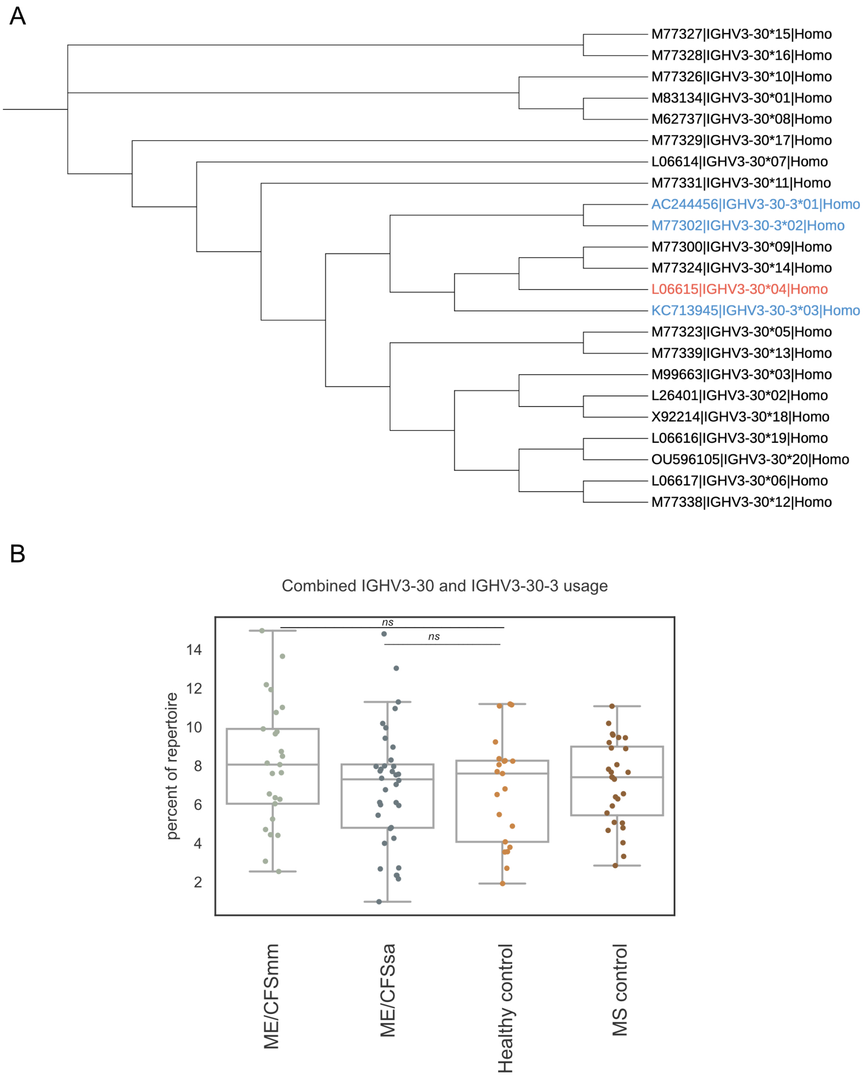

Supplementary Figure 2:
A) Neighbour joining tree of all available IGHV3-30 and IGHV3-30-3 alleles in the IGMT reference database. IGHV3-30-3 highlighted in blue. IGHV3-30*4, which has an identical sequence to IGHV3-30-3*03, is highlighted in red. Reference sequences were obtained from IGMT: https://www.imgt.org/vquest/refseqh.html#VQUEST. **B)** V gene usage for combined IGHV3-30 and IGHV3-30-3.

Figure S2.
